# Editorial: From brain priorities to brain modeling

**DOI:** 10.3389/fpsyt.2023.1272054

**Published:** 2023-10-16

**Authors:** Orestis Giotakos

**Affiliations:** Independent Researcher, Athens, Greece

**Keywords:** intentionality, predictive coding, natural language processing, functional neurological disorder, unexpected stimulus omissions, brain modeling, mental health, computational psychiatry

Brain modeling derived from the brain priorities is expected to make scientific communication more precise, leading to applications across science, health care, and technology. Grounded in clinical neuroscience and guided by brain modeling, psychiatry can improve both assessment and treatment strategies. In this way, psychiatry can develop more accurate interventions, via a deeper understanding of genetics, pathophysiology, functional neuroanatomy, and neuropsychopharmacology.

Things have been intentionally designed by purposive minds, while the mind is in good working order to the extent that its intentional objects and connections are appropriate (1). Moreover, phenomenology suggests affective intentionality as an embodied and enactive process that connects us to a shared world and guides our dealings with it. Emphasizing the intentional stance of things, the paper of Giotakos proposed an “intentional system”, as a higher order system, with a monitoring and regulatory role attributed to the brain and behavior. A series of potential associations have been presented, by observing the conceptual and neurobiological traces of the intentionality main features of “aboutness” and “directedness”. A permanent failure of intentionality dominates in psychosis, because of an inappropriateness of the intentional object or connection. Affective disorders may result from imprecise interoceptive prediction error signals, due to a confused identification of the intentional object, while in suicidal patients there is an emotional intentionality failure, characterized by an absence of intentional object or a loss of conscious access to normal intentional objects.

As a predictive organ, the brain's overall function is to minimize long-term average prediction error. Predictive coding is a neurocognitive concept, according to which the brain does not process the whole qualia of external information, but only residual mismatches occurring between incoming information and an individual, inner model of the world, thus minimizing the free energy or brain entropy (2). An innovative approach to studying surprise is through the use of stimulus omissions, where expected standard stimuli are occasionally replaced by an unexpected stimulus absence to elicit surprise. In this Research Topic, the paper of Dercksen et al. investigated the pupil's response to unexpected stimulus omissions in order to better understand surprise and orienting of attention resulting from prediction violation. This study offers a robust and unconfounded approach for investigating the neural mechanisms of surprise. This pupillometry approach is especially suitable to study sensory prediction in vulnerable populations within the psychiatric field. The findings demonstrate predictive models in brain processing and point to the involvement of subcortical structures in the omission response.

In another field of neuropsychiatry, although functional neurological disorder (FND) are neurological disorders not explained by currently identifiable histopathological processes, the pathophysiology is reflective of dysfunction within and across different brain circuits that, in turn, affects specific constructs, i.e., salience, multimodal integration, and attention networks (3). Functionally impaired awareness disorders (FIAD) encompass a range of subjectively altered awareness, ranging from focal sensory disturbance to profound global disorders such as trance states and functional coma. A computational perspective is highly consistent with *models of illness* structuring FND. In this Research Topic, Milano et al. give an overview for the functionally impaired awareness disorders (FIAD), able to guide both research priorities and diagnostic formulation. The authors proposed that aspects such as the role of learning *via* stress and trauma, attention modulation, functional and effective connectivity, and the computational role of different levels of brain hierarchy need to be further investigated.

On the other hand, major improvements in neural networks and deep learning architectures have enabled the transition from statistical to more complex and flexible models. The machine learning algorithms are already used for the detection and diagnosis of bipolar disorder (4) and psychosis (5). The potential value of linguistic biomarkers has been significantly magnified by recent advances in natural language processing (NLP) and machine learning. Although its applications to neuroscience and psychiatry are still in their infancy, NLP is used for many different purposes in neuroscience and psychiatry (6). In this Research Topic, the paper of Crema et al. give an overview for the ability to automatically analyze any type of medical document artificial intelligence among 1,024 papers that used NLP technology in neuroscience and psychiatry from 2010 to early 2022. One hundred and fifteen papers were evaluated. First, the paper shows that NLP is used for many different purposes in neuroscience and psychiatry. NLP could be an effective tool to help clinicians gain insights from medical reports, clinical research forms and more, making NLP an effective tool to improve the quality of healthcare services. The authors concluded that the establishment of minimum requirements, further standardization, and external validation will likely soon increase the prevalence of NLP applications in neuroscience and psychiatry.

Course of disease is probably the most crucial aspect of disease burden in patients suffering from Major Depressive Disorder (MDD). Incorporating multiple clinical variables of MDD disease course and linking these measures to brain morphometric alterations in different samples will provide reliable measures in clinical research and practice (7). In order to understand the historical growth, current state, and potential expansion trend, the paper of Wang et al. used the natural language processing (NLP) method and multiple algorithms, such as Latent Dirichlet allocation (LDA), to conduct a bibliometric analysis of 5,566 publications related to depression and radiology in the past 20 years. This study hopes to extract relevant research focus, discover changes in research content, predict possible future breakthroughs direction, and summarize the research of the last years. This is the first such analysis in this field. It was found that recent research focuses are “Symptoms and sleep,” “Brain structure study,” and “functional connectivity. The most research on brain structure were extracted, among which Cerebral Cortex, Prefrontal Cortex, and Hippocampus are the most studied. High citation review mainly covers the mechanism of acute injury, inflammation, genetic factors, trauma, vascular microembolism, ischemia, and migraine model on MDD, and also includes the criteria and discussion of systematic review methods. Finally, the results suggest that the most cited publication can be roughly divided into two categories, one focuses on the establishment of diagnostic criteria for depression and the other on the detection of MDD brain regions in radiology methods, such as the cerebral cortex and the Large-Scale Network Dysfunction.

The goal of this Research Topic is to strengthen the dialogue, in order to fill the gap between neuroscience knowledge and the mental health unmet needs. Creating brain modeling, computational neuroscience employs mathematical models and abstractions of the brain to understand the principles that govern the development and the priorities of the brain. For this reason, in this Research Topic, titled *From brain priorities to brain modeling*, we provide a collection of compelling research studies and reviews, which brings together advances in both neuroscience and machine learning methods.

## Author contributions

OG: Conceptualization, Writing—original draft.
